# The Emerging Role of MicroRNAs in Hepatocellular Carcinoma

**DOI:** 10.5005/jp-journals-10018-1095

**Published:** 2014-01-22

**Authors:** Hikmet Akkiz

**Affiliations:** Department of Gastroenterology, Qukurova University, Adana, Turkey

**Keywords:** Hepatocellular carcinoma, MicroRNA, Oncogene, Tumor suppressor gene.

## Abstract

Hepatocellular carcinoma (HCC) is common malignancy and a leading cause of cancer death worldwide. Recent epidemiological data have demonstrated that liver cancer incidence is continuously rising and will continue to do so for more than a decade, not only South Africa and Mainland China but also in North America and Europe. Molecular profiling of changes in gene expression has improved our understanding of the HCC mechanism. MicroRNAs (miRNAs) are a class of small regulatory RNAs that function to modulate protein expression. This control allows for fine-tuning of the cellular phenotype, including regulation of proliferation, cell signaling and apoptosis. Recently, investigators have demonstrated decreased or increased expression of particular miRNAs in hepatobiliary cancer cells. Many studies have highlighted the role of miRNA in physiological processes and cancer development. Several studies have reported that some miRNAs may play a role in the development and progression of HCC. Recent investigations have suggested that the presence of single nucleotide polymorphisms in miRNA genes, their processing machinery and target binding sites affect cancer risk, treatment efficacy and patient prognosis. This review will discuss the emerging critical role of miRNAs in hepatocarcinogenesis, HCC progression and clinical outcome.

**How to cite this article:** Akkiz H. The Emerging Role of MicroRNAs in Hepatocellular Carcinoma. Euroasian J Hepato-Gastroenterol 2014;4(1):45-50.

## INTRODUCTION

Hepatocellular carcinoma (HCC) is the fifth most common cancer and the third leading cause of cancer-related death worldwide.^1^ Although most cases occur in Asia and Africa, the incidence has been steadily increasing in the west over the last 20 years.^2,3^ Chronic hepatitis B and C infections as well as chronic alcohol use are the most common risk factors worldwide.^3^ Despite great advances in the treatment of HCC, the 5-year survival rate remains quite low among patients with HCC.^4^ Surgical resection and liver transplantation are currently the best curative options to treat HCC.^5^ However, only 5 to 15% of HCC patients are currently eligible for surgical intervention, based on the evaluation of their liver function and tumor burden.^6^ Moreover, recurrence and metastasis is common in patients who have had a resection, and postoperative 5-year survival is only 30 to 40%.^7^ Current data have demonstrated that there is an urgent need to develop molecular tools in assisting early HCC diagnosis, prognosis and treatment strafication.^8^

The development and progression of HCC is typically a multistage process and develops usually in cirrhotic liver.^9^ The transforming begins in the liver tissue undergoing chronic hepatitis or cirrhosis. Progresses through a series of hyperplastic and dysplastic stages, and ultimately acquires the malignant phenotype with intrahepatic metastasis and distal dissemination.^10^ Our understanding of HCC has been improved by the recent studies on molecular profiling to identify changes in gene expression that are associated with particular phenotype, such as HCC subtypes, recurrence or metastasis.^11^ Tumor biology based on molecular analyses can provide a more accurate assessment in prognosis than conventional pathology. For example, serum alpha-fetoprotein (AFP) is a useful biomarker for HCC diagnosis.^12^ Yamashita et al recently published an article addressing epithelial cell adhesion molecule (EpCAM), a new marker for cancer stem cells (CSCs) in HCC. They found a population of HCC cells expressing EpCAM, an epithelial cell adhesion molecule previously identified as a marker for stem progenitor cells of adult liver and oval cells.^13,14^ Yamashita et al have also demonstrated that EpCAM expression in tumors with serum AFP can predict two distinct prognostic HCC subtypes, i.e. EpCAM (+) AFP (+) HCC (referred to as HpSC—HCC; hepatic stem cell—like HCC) with poor outcome and EpCAM (-) AFP (-) HCC, (referred to as MH-HCC; mature hepatocyte—like HCC) with good prognosis.^13,14^ More recently, a study published by Terris et al suggest that HCC growth and invasiveness is dictated by a subset of EpCAM (+) cells.^15^

In mammalian cells, protein-coding RNAs account for 5% of the total RNA population.^16^ Several species of noncoding RNAs with regulatory functions have emerged in the recent years. Among these, a new class of RNAs, the miRNAs, has been discovered and their aberrant expression has been linked to the pathogenesis of many cancers due to their ability to regulate the expression of crucial RNAs.^17^ MiRNAs are noncoding RNAs that regulate both mRNA and protein expression of target genes.^18,19^ The description and regulation of miRNA biogenesis has been extensively reviewed.^20-22^ Despite the emerging critical role of miRNAs, the mechanism of their action is yet to be fully understood. Transcription of miRNA genes is under control of promoter elements regulated by established transcription factors, such as c-Myc.^23,24^ This regulation of expression may provide for clinically useful point of intervention, either by stimulating a miRNA whose expression is inappropriately suppressed or by inhibiting expression of an amplified miRNA.^18^ The primer transcript is cleaved by the endonuclease-containing microprocessor complex in the nucleus to yield the precursor miRNA.^25^ Of note, increased processing of miRNA-21 (mir-21) primary transcript by transforming growth factor beta (TGF-b)-induced SMAD activity has recently been described in vascular smooth muscle cells.^26^ Surprisingly, the mechanism is through the noncanonical action of SMAD binding to the RNA helicase p68 rather than transcriptional activation.^27^ It remains to be seen if SMAD-assisted processing contributes to mir-21 overexpression in hepatobiliary cancers, or if SMAD increases processing of other primary miRNAs.^28^ The precursor miRNA is then exported from the nucleus and cleaved in the cytoplasm by Dicer, and the mature miRNA is incorporated into the RNA-induced silencing complex (RISC) by the RISC-loading complex.^18^ The resulting miRNA-loaded RISC contains a single-stranded miRNA 19 to 23 nucleotides in length that guides sequence-specific translational supression.^17,28^ Silencing of miRNA targets is directed by base-pairing of the miRNA to the cognate messenger RNA (mRNA) ‘seed’ nucleotides 2 to 7. Augmented by neighboring nucleotides. Thus, miRNAs have dozens to hundreds of targets.^18,29^

Recently, the discovery of aberrantly expressed miRNAs in HCC further improved our understanding of this disease. Similar to mRNA, HCC-associated miRNAs could be used as diagnostic and prognostic biomarkers of HCC with a potential for even greater accuracy.^30^ It is generally observed that miRNA expression levels are decreased in cancer tissue compared to nontumor tissue.^31^ This may be attributable to a broad suppression of miRNA expression by cancer-associated transcription factors. For example, c-Myc suppresses expression of more than 10 miRNAs in two B-cell lymphoma models.^25^ Alternatively, in cancer cell lines, precursor miRNAs were present in the nucleus, but the mature form was absent from the cytoplasm, including liver tissue and HCC samples.^32^ This suggests post-transcriptional regulation, presumably by altered degradation of the mature miRNA remains a possibility.^18^ In a rat model of induced HCC, mir-122 expression was decreased, a finding also observed in 50% of human tumor sample.^33^ This finding is also observed in 50% of human tumor samples.^33^ In HCC, an array-based analysis identified 44 miRNAs that were expressed at lower levels in HCC compared to normal livers.^34^ Separate study comparing HCC to liver cirrhosis demonstrated downregulation of 34 miRNA in HCC.^17^ These data suggest decreased expression of the liver-specific miRNA, miR-122, as well miR-199.^18^

Tumor suppressors are often lost through genetic or epigenetic mechanisms, but silencing through miRNA targeting may also be important. For example, phosphatase and tensin homolog deleted on chromosome 10 (PTEN) is a tumor suppressor that counters phosphatidylinositol 3 -kinase (P13K) activation.^18^ P13K stimulates AKT in a survival signal; thus, PTEN silencing allows P13K AKT activation and inappropriate survival of cancer cells.^18^ Loss of PTEN allows unchecked cell cycle progression. PTEN has been shown in HCC as a target for mir-21, which is frequently upregulated in cancer.^34^

Several studies have started to investigate for specific miRNA deregulation in hepatitis B virus (HBV)-related and hepatitis C virus (HCV)-related HCCs.^35^ Gene encoding miRNAs have been found in viruses and viral miRNAs have a regulatory effect on their protein-coding genes.^36^ This regulatory effect may be beneficiary to the virus toward maintaining its replication, latency and evading the host immune system.^37^ miRNAs from the host cells may play a role in building up direct or indirect effect in regulating viral genes.^38^ miR-122 is first identified liver-specific cellular miRNA, which has been shown to enhance the replication of HCV by targeting the viral 5’ noncoding region.^37,38^ Indeed, HCV RNA can replicate in Huh 7 cells, which express miR-122, but not in HepG2 cells, which do not express miR-122.^36^ Additionally, silencing of miR-122 in hepatocyte resulted in a marked loss of replicating HCV RNAs.^37^ A putative miR-122 binding site in the 50-end of HCV genome was identified, suggesting a direct role of miR-122 in HCV replication.^39^

Currently, there are some controversies about the role of miR-122 in HCV infection. A study published by Henke et al^40^ showed that miR-122 stimulates HCV translation by enhancing the association of ribosomes with viral RNA. The findings describing the role of miR-122 in HCV replication are of special interest, not only because of its novel mode of action, but because they also provide the first evidence of miRNAs linked to infectious disease.^31^ The development of therapeutic products to inhibit the miR-122 is believed to be an attractive approach to treat HCV patients.^31^

By contrast, a study published by Sarasin-Filipowicz et al^41^ has not shown a correlation between miR-122 expression and viral load in patients receiving interferon therapy. Moreover, they found markedly decreased miR-122 levels in patients who failed virological response. These reports indicate that the role of miR-122 in HCV viral replication is still controversial and awaits future research.

While some host miRNAs are beneficial for the virus, others inhibit viral replication. Studies published by Pedersen et al^42^ indicated that interferon B (IFN-B) induces several cellular miRNAs, specifically eight miRNAs that have sequence-predicted targets within the HCV genomic RNA. Overexpression of these miRNAs in infected liver cells considerably attenuated viral replication.^42^ From this report, it seems that host miRNAs have evolved to target viral genes and inhibit their replication, and thus might represent of the host antiviral immune response.^31^ Most recently, Peng et al^43^ have found differential profiles of cellular miRNAs that target the genes involved in chemokine, B-cell receptor, PTEN, IL-16, ERK MAPK and JAK STAT signaling pathways, suggesting a critical role of miRNAs in the replication, propagation, and latency of virus in the host cell. They have also demonstrated that miR-122, miR-320 and miR-191 were downregulated, whereas miR-215, miR-16, miR-26, miR-130, miR-199 and miR-155 are upregulated.^43^ These findings suggest that miRNAs have potential to become novel drug targets in virally induced infectious or malignant disease.^31^

A differential miRNAs expression pattern was found in the livers of HBV and HCV-infected patients with HCC.^44^ A total of 19 miRNAs were clearly differentiated between HBV and HCV groups, out of which 13 miRNAs were down-regulated in HCV group, whereas six showed a decreased expression in HBV group.^44^ Some of the differentially regulated miRNAs between the HCV and HBV groups were miR-190, miR-134, miR-151, miR-193, miR-211 and miR-20. Same group has also demonstrated that pathway analysis of targeted genes using infection-associated miRNAs could differentiate the genes into two groups. For example, in HBV-infected livers, pathways related to cell death, DNA damage, recombination and signal transduction were activated, and those related to immune response, antigen presentation, cell cycle, proteasome and lipid metabolism were activated in HCV-infected livers.^44^

## MICRORNAs AND HEPATOCELLULAR CARCINOMA

Several studies have shown that specific miRNAs are aberrantly expressed in malignant HCC cells or tissues compared to nonmalignant hepatocytes or tissue.^45^ HCC subtypes have also been shown to have distinct miRNA profiles, allowing one to distinguish whether it is CSC-like or mature hepatocyte-like, malignant or benign, metastatic or nonmetastatic, viral or nonviral in origin, and whether it is caused by HBV or HCV.^46^ Using microarray technologies, Murakami et al^47^ first profiled miRNA gene expression in 24 HCCs and 22 non-HCC liver tissues, and found that eight miRNAs were differentially expressed. Following this study, several investigators have also identified miRNA signatures that differentiated cancerous from adjacent, noncancerous liver tissues.^48,49^ Another group developed a miRNA signature using HCC and normal liver tissues.^50^ By comparing the miRNAs profiles between cirrhotic liver and HCC tissues, Gramantieri et al^17^ also found that 35 miRNAs including miR-122 differ between HCC and cirhosis. New study published by Budhu et al^51^ in 241 HCC cases revealed that one-third of available miRNAs were significantly altered in HCC. However, the expression of these miRNAs was heterogeneous, indicating that different miRNAs are associated with unique tumor biology and distinct HCC outcomes.^46^

Growing data have suggested that aberrantly expressed miRNAs may work as functional actors in HCC initiation and progression.^46^ Selected miRNAs, such as miR-21, miR-224, miR-34a, miR-221 222, miR-106a, and miR-203 are upregulated in HCC compared to benign hepatocellular tumors, such as adenomas or focal nodular hyperplasia.^39,45^ Certain miRNAs have been reported to be decreased in HCC compared to nontumoral tissues, such as miR-122a, miR-422b, miR-145 and miR-199a.^39,45^ Liver-specific miR-122 is significantly downregulated in HCC tissues.^46^ It functions as a potential tumor suppressor in two ways: inhibiting hepatic cell growth by targeting cyclin G1 and promoting apoptosis of hepatic cells by targeting Bcl-w. Additionally, miR-122 also plays a positive role in HCC development by stimulating HCV RNA translation. Besides miR-122, most miRNAs with altered expression level in HCC are involved in promoting loss of cell cycle control in HCC.^52,53^

In human cancer, miRNAs can function as oncogenes or tumor-suppressor genes during tumorogenesis and progression. More recently, some specific miRNAs were found to be associated with the clinicopathological features of HCC, such as metastasis, recurrence and prognosis.^54,55^ Invasion and metastasis are leading lethal factors for cancers. The long-term survival rate of HCC patients after curative resection is still low because of high recurrence rate.^56^ Identification of metastatic factors and understanding the mechanisms underlying metastasis are important for treatment of HCC. Meng et al^34^ reported that aberrant expression of miR-21 cannot only contribute to HCC growth but also mediate HCC cell invasion by direct targeting PTEN. Recently, PTEN was found to be direct target of miR-221 and miR-222, which induce TRAIL resistance and enhance HCC cell migration.^57^ miR-221 and miR-222 also regulate the expression protein phosphatase 2A subunit B and TIMP3 tumor suppressors, thus activating the AKT pathway and metallopeptidase to promote HCC cell invasion and meta-stasis.^58^ Some investigators reported that miR-30d and miR-151 involved in HCC invasion and metastasis.^59,60^ The miR-30d is frequently upregulated in HCC and its expression is associated with intrahepatic metastasis.^60^

The miR-122 is significantly downregulated in liver cancers and inhibits HCC intrahepatic metastasis by regulation of a disintegrin and metalloprotease family protein ADAM10 and ADAM17.^61,62^ The hepatocyte growth factor (HGF) c-Met signaling cascade may involve in HCC metastasis. The HGF interacts with c-Met receptor tyrosine kinase and leads to invasive growth by stimulating invasion and protection from apoptosis.^56^ c-Met oncogene is associated with the aggressive nature and the poor clinical outcomes of many tumors such as HCC.^56^ c-Met oncogene is regulated by miR-1, miR-34a, miR-23b and miR-199a-3p. All of them are downregulated in HCC.^56^ Silencing of miR-1 can not only inhibit HCC growth but also mediate HCC cell invasion by downregulating c-Met.^63^ The miR-34a inhibits tumor cell migration and invasion by decreasing c-Met-induced phosphorylation of extracellular signal-regulated kinases 1 and 2.^64^ The miR-23b also inhibits the migration and proliferation ability of HCC cells by downregulating c-Met and urokinase-type plasminogen activator.^65^ miR-199a-3p induced G1 phase cell cycle arrest and reduced invasive capability by targeting c-Met and mTOR.^66^

Sequencing has shown that single nucleotid polymorphisms (SNPs) in miRNA coding genes, and specifically in miRNA seed regions, are rare.^67^ SNPs in miRNA genes are thought to affect function in one of three ways: first, through the transcription of primary transcript; second, through pri-miRNA and pre-miRNA processing and, third, through effects on miRNA-mRNA interactions.^19^ A study published by Akkiz et al^68^ demonstrated that a functional polymorphism in pre-miRNA-196a-2 contributes to the susceptibility of HCC. On the other hand, the same group showed that the miR-146a polymorphism has no role in genetic susceptibility to hepatocarcinogenesis.^69^

## CONCLUSION AND FUTURE PERSPECTIVE

Current findings demonstrate that miRNAs are aberrantly expressed in HCC. Some miRNAs are involved in hepato-carcinogenesis by promoting stemness of CSC and by controling cell proliferation and apoptosis; others are associated with HCC progression by controlling cell migration and invasion. Similar to protein coding genes, miRNAs are transcribed by RNA polymerase II and may be regulated at transcriptional levels. miR-181 is regulated by TGF-B signaling; miR-143 is regulated by NF-kB; miR-221 and miR-222 are regulated by HGF c-Met signaling through the c-jun transcription factor. Genetic and epigenetic aberrations may also contribute to the deregulation of miRNAs in HCC.

Hepatocellular carcinoma-associated miRNAs not only provide new insights into the molecular basis of HCC but also serve as new biomarkers for HCC diagnosis and prognosis. Aberrantly expressed miRNAs associated with specific biopathological and clinical features can establish the basis for the development of a more rational system of HCC classification and therapeutic approaches.
